# Domain Structures in Nematic Liquid Crystals on a Polycarbonate Surface

**DOI:** 10.3390/ijms140816303

**Published:** 2013-08-07

**Authors:** Alexander M. Parshin, Vladimir A. Gunyakov, Victor Y. Zyryanov, Vasily F. Shabanov

**Affiliations:** Kirensky Institute of Physics, Russian Academy of Sciences, Siberian Branch, Krasnoyarsk Scientific Center, Krasnoyarsk 660036, Russia; E-Mails: gun@iph.krasn.ru (V.A.G.); zyr@iph.krasn.ru (V.Y.Z.); dir@iph.krasn.ru (V.F.S.)

**Keywords:** liquid crystal, polymer structure, domain

## Abstract

Alignment of nematic liquid crystals on polycarbonate films obtained with the use of solvents with different solvations is studied. Domain structures occurring during the growth on the polymer surface against the background of the initial thread-like or schlieren texture are demonstrated. It is established by optical methods that the domains are stable formations visualizing the polymer surface structures. In nematic droplets, the temperature-induced transition from the domain structure with two extinction bands to the structure with four bands is observed. This transition is shown to be caused by reorientation of the nematic director in the liquid crystal volume from the planar alignment to the homeotropic state with the pronounced radial configuration of nematic molecules on the surface. The observed textures are compared with different combinations of the volume LC orientations and the radial distribution of the director field and the disclination lines at the polycarbonate surface.

## 1. Introduction

The study of structural ordering of liquid crystals (LCs) is of great importance for both the development of physics of condensed matter and application. There exist a few general LC structures. First of all, it should be noted that, on untreated solid substrates in the absence of polar forces, nematic LC molecules align parallel to one another and bounding surfaces. In this case, we deal with an inhomogeneous orientation smoothly varying over the substrate plane. Depending on an LC layer thickness, either thread-like or schlieren texture is observed [1,2]. The former consists of homogeneous mesophase regions separated by movable linear disclinations; the latter has singular points with outgoing lines (distorted regions of the planar orientation). In these textures, defects are the nematic medium objects that obey topological laws.

Another type of the structure is local formations in the volume or on the surface of a nematic LC. The most typical of them are nematic droplets floating in an isotropic liquid [3]. They arise in the form of a dispersive phase distributed in a dispersive medium with the formation of a dispersive system. Similar droplets form at the decay of a three-component system LC-solvent-polymer [4]. Nonuniform orientation of the nematic director in droplets is caused by the boundary conditions (normal or tangential) and the orientational anisotropy of the LC elastic free energy. Configuration of the nematic director is completed on point defects (one in the center and two on the poles of a droplet), which are locally restricted and do not interact with one another and defects of neighboring droplets. The conditions for the existence of droplets are more complex when the latter are located on the LC surface, e.g., the nematic-isotropic liquid (NI) interface [5]. Varying the temperature, one can change the position of the interface in an LC cell; then, the director configuration will be additionally affected by external factors (bounding surfaces of LC cells, forces of gravity, *etc*.). Under such conditions, point defects interact with one another as on the untreated solid surfaces. Placing droplets on water, one can obtain stable local formations of a nematic LC on the liquid surface [6]. The objects arising in this case have the form of hemispheres (lenses) suspended to the water boundary. The lenses with two surface defects at the diameter ends are stable and independent of one another and external factors. Study of the LC behavior on non-solid boundaries are of interest, since such boundaries make it possible to observe a regular network of point defects that can arise, e.g., on a free nematic LC surface. The network consists of domains formed by protrusions and troughs where the nematic director configuration is determined by the competing effects of elastic, surface, and gravity forces [7]. The similar network arose at the NI interface [8] and in hybrid ordered thin (~1 μm) nematic films with a liquid (glycerol or polyethylene glycol) surface [9].

One more type of the structure can arise in a nematic LC layer with the orienting centers occurring during the transition of the metastable isotropic phase to the stable nematic one. As was demonstrated in [10], upon gradual cooling of nematics from an isotropic liquid on the polyimide surface, nucleation occurs either in the volume of a nematic LC or on its surface, depending on polymer polarity. In such systems, under the temperature variation, spherical domains spontaneously occur that grow in time. The domains are stable and do not interact with one another. The kinetics of the phase ordering during the domain growth was studied in [11].

In this study, we investigate the alignment of nematic LCs on polymer surfaces in the presence of different solvents. We demonstrate that, on polymer structural elements, a network of stable domains arises, which can be considered as a superposition of three different configurations of the director.

## 2. Results

**Figure 1:**
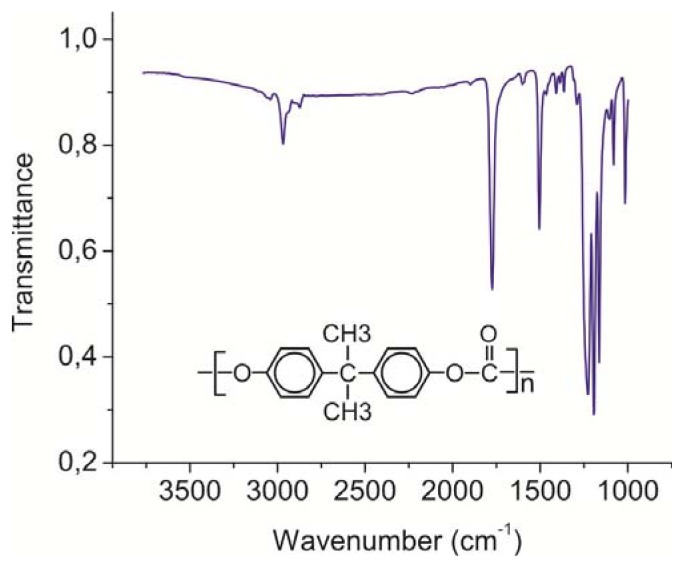
IR absorption spectrum of the thin polycarbonate (PC) film dried from the dichloromethane solution deposited onto the CaF_2_ surface.

The principal peaks in the PC film IR spectrum shown in Figure 1. They should be caused by the following vibrations: C–H aromatic ring deformations around 3000 cm^−1^; C=O carbonate group deformations near 1775 cm^−1^; C=C-vibrations at 1506 cm^−1^; asymmetric O–C–O carbonate group deformations in the range 1232–1164 cm^−1^; CH_3_-vibrations at 1081 cm^−1^; symmetric O–C–O carbonate group deformations near 1015 cm^−1^ [12].

### 2.1. LC Domains on the PC Surface

**Figure 2:**
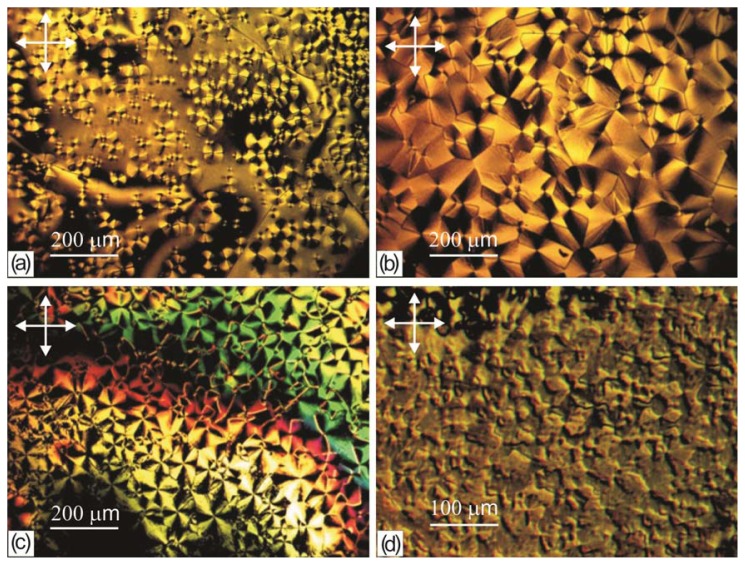
Microphotographs of the 5CB domain textures on the surface of a film obtained from the PC solution in (**a**–**c**) dichloromethane and (**d**) pyridine: (**a**) b-domains in an liquid crystal (LC) droplet after growing for 5 min; (**b**) b-domain ensemble in an LC droplet after the 15 min growth finish; (**c**) c-domain ensemble at the droplet edge in 15 min; and (**d**) island domains in an LC droplet. Arrows show the polarizer directions.

In first seconds, in a sufficiently thick LC layer (δ > 10 μm) on the PC film we observed the initial thread-like texture with homogeneous regions and linear disclinations. In thin layers (δ < 10 μm), we observed the schlieren texture with point defects and dark bands. In several tens of seconds, butterfly-shaped domains (b-domains) spontaneously arose on the PC surface against the initial LC texture background. The domains arose randomly and grew sequentially, one after another, with a rate depending on a deposition rate and a time of drying the polymer film prior to the LC deposition (from few seconds to several days). The b-domain growth against the thread-like texture background is illustrated in Figure 2a. When growing, the domains form an ensemble (Figure 2b) resembling the polygonal texture in smectics [1]. In the thin layer (δ < 6 μm) at the LC droplet edge one can observe the cross-shaped domains (c-domains) forming a regular network (Figure 2c). The c-domain ensemble is revealed as a group of colored stripes with the colors corresponding to the interference spectra of the wedge-shaped nematic layer in a droplet. The LC domain textures were also observed in the cells with a cover glass; in contrast to [7], the domain network was independent on forces of gravity, since the domains arose at an arbitrary spatial position of the cell. The domains were stable formations tightly bound to the surface and did not change their internal configuration during the growth. When pyridine is used as a solvent, the nematic texture consisting of islands without point or linear defects was observed on the PC surface (Figure 2d).

**Figure 3:**
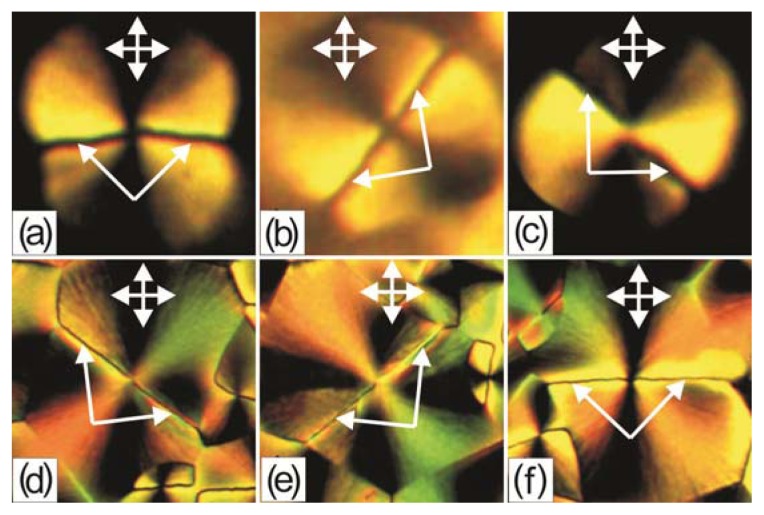
Microphotographs of 5CB domains on the PC film obtained from the solution in dichloromethane: (**a**) b-domain with the disclination lines beyond the extinction bands; (**b**) b-domain upon rotation of a sample by an angle of about 45°; (**c**) b-domain with the disclination lines beyond the extinction bands; (**d**) c-domain with the disclination lines beyond the extinction bands; (**e**) c-domain upon rotation of a sample by an angle of 90°; and (**f**) c-domain with the disclination lines passing through the centers of the horizontal extinction bands. Arrows show the disclination lines and light polarization directions.

Figure 3 presents microphotographs of two individual b- and c-domains in crossed polarizers. It can be seen that the b-domain is a disk with two extinction bands when the disclination lines are beyond them (Figure 3a). When the sample is rotated, the forms of the domain and the disclination lines do not change but the background above the domain brightens (Figure 3b). If the disclination lines in the domain appear near the dark and bright domain region boundaries, then one can observe the brightened sectors (Figure 3c). The c-domain is cross-shaped with four extinction bands when the disclination lines are beyond them (Figure 3d). When the sample is rotated by an angle close to 45°, the forms of the domain and disclination lines do not change and the extinction bands stay at their position (Figure 3e). However, if the disclination lines appear close to one of the light polarization directions, the corresponding extinction bands brighten (Figure 3f).

**Figure 4:**
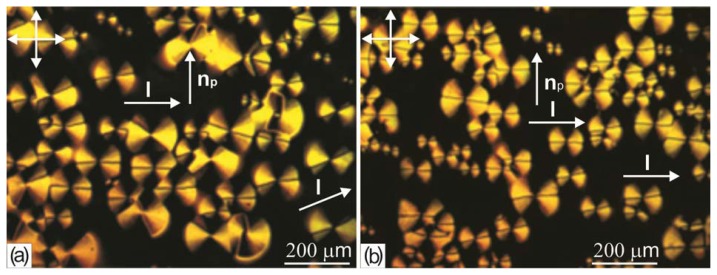
Microphotographs of the 5CB b-domain ensemble arisen during the growth on the surface of the PC film obtained from the solution in dichloromethane in a cell with the planar layer thicknesses (**a**) δ = 10 and (**b**) 6 μm. Arrows show the directions of light polarization, preferred orientation of the disclination lines l, and nematic director n_p_ in the cell volume.

Study of the structural ensembles during the growth in the LC droplets showed that the disclination lines in domains tend to orient perpendicular to the director of the volume nematic layer located above the domains. In the thin LC layer with the schlieren texture, the lines mainly follow the director orientation, while in the thick layer with the thread-like texture they can locally change the nematic alignment near a domain. The effect of the ordering of the disclination lines by the director of the LC layer above the domains was unambiguously revealed in the cells with a cover glass rubbed with silk cloth to form the homogeneous planar orientation of the nematic. In Figure 4a, one can see the trend of the disclination lines to align in the l direction perpendicular to the nematic director n_p_ in the cell with the planar layer thickness δ = 10 μm. As the layer thickness is decreased to δ = 6 μm (Figure 4b), the almost complete orientation l ⊥ n_p_ is implemented over the entire cell. Microphotographs similar to those shown in Figures 2, 3 and 4 were obtained for MBBA.

### 2.2. Domain Growth

**Figure 5:**
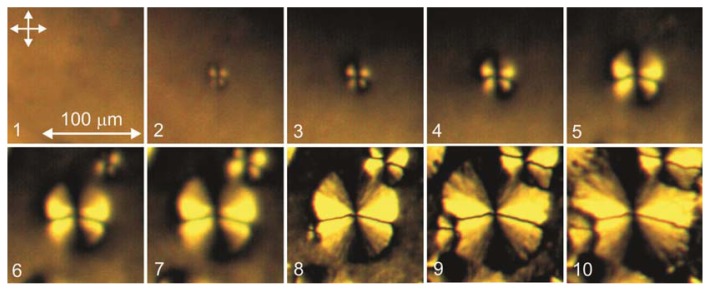
1-min-interval frames of a growing 5CB domain deposited in the form of a droplet on the PC film obtained from the solution in dichloromethane.

The b-domain growth is illustrated in Figure 5. The domain arises from nucleation seeds and grows to the finite size d ~ 170 μm. During the domain growth, a new nucleus (Frame 6) arises nearby and grows together with the domain. The disclination lines extend with the domain radius. In the case under consideration, they are close to the analyzer direction. In the LC volume, the director makes a certain angle with this direction; therefore, in crossed polarizers the layer looks bright.

**Figure 6:**
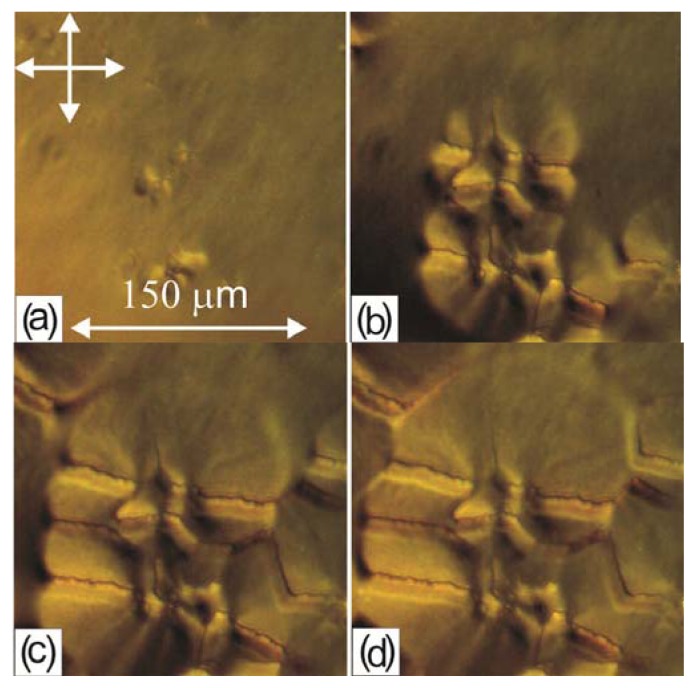
3-min-interval frames of a growing MBBA domain deposited in the form of a droplet on the PC film obtained from the solution in dichloromethane: (**a**) initial state; (**b**) in 3 min; (**c**) in 6 min; (**d**) in 9 min.

Figure 6 shows the growth of the group of MBBA b-domains arisen next to each other. The disclination lines of all the domains are not extended as those of the b-domain in Figure 5 but have a zigzag shape. In the domain centers, dark defect regions are observed.

**Figure 7:**
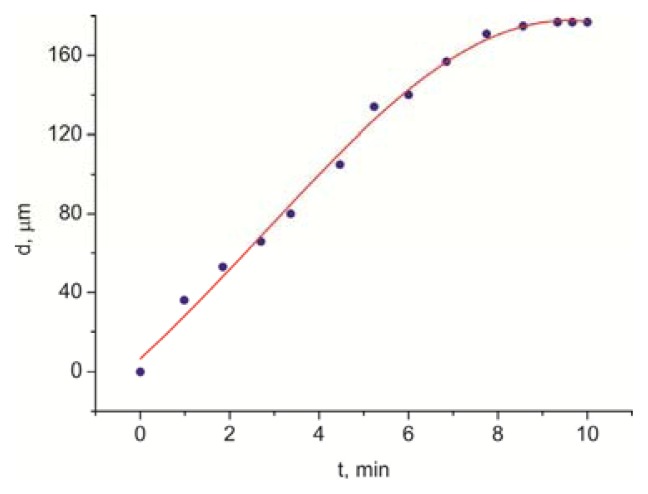
Time dependence of diameter *d* of a growing 5CB domain deposited on the PC film obtained from the solution in dichloromethane in 1 min (dots). Solid line is the interpolation.

Figure 7 shows the dependence of domain diameter *d* on growth time *t*. The dependence is linear up to reaching the finite size.

### 2.3. Temperature-Induced Orientational Transition

**Figure 8:**
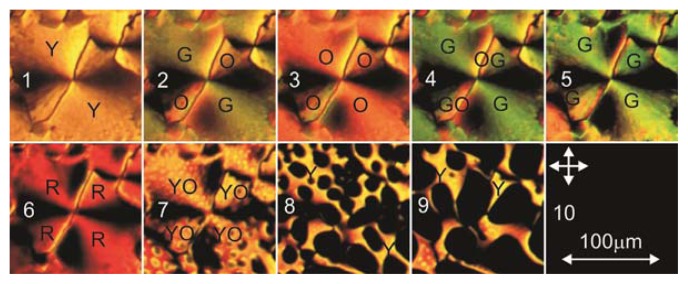
Frames 1–10 reflecting the changes in the MBBA layer texture on the surface of the film obtained from the PC solution in dichloromethane at the temperatures *T* = 35, 35.4, 35.8, 41, 41.3, 41.6, 42, 43, 45 and 47 °C, respectively. Y, O, G, and R denote yellow, orange, grey, and red colors. Arrows show the light polarization directions.

Figure 8 presents texture transformations in the MBBA cell with the PC film that occur at temperature variations. In the range from 21 to 35.8 °C (Frame 3), the LC texture does not change. As the temperature is increased to *T* = 41.6 °C (Frame 6), the interference variation of the image color is observed. Simultaneously, in the b-domain with two extinction bands, two additional extinction bands gradually arise and the b-domain transforms to the c-domain with four extinction bands. These bands are clearly seen, since the disclination lines in the investigated domain make an angle close to 45° relative to the light polarization directions and do not brighten the image. However, the change in the visualized *c*-domain structure in the temperature range under consideration is not observed. Therefore, we may conclude that the orientational temperature transformations occur in the volume LC layer above the domains at the reorientation of the director from the planar to homeotropic structure. At a further increase in the temperature to *T* = 47 °C, the c-domain gradually vanishes, which corresponds to the transition of the entire LC layer to the isotropic phase (Frame 10).

### 2.4. Effect of the Residual Solvent in the PC Film on the LC Structure

**Figure 9:**
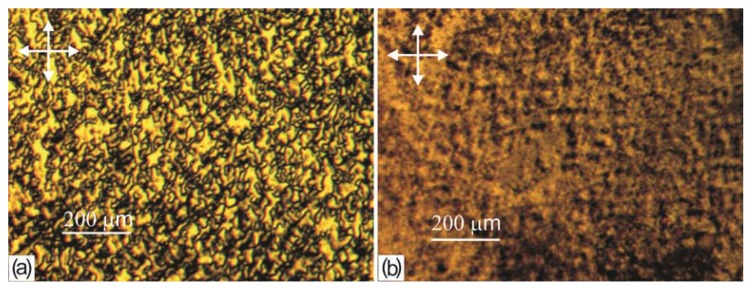
Microphotographs taken in 4 h from the 5 CB textures on the surface of the PC film obtained from the solution in (**a)** chloroform and (**b**) pyridine and dried at the temperature *T* = 120 °C for 15 min.

In our experiments, we used granular PC dried at the temperature *T* = 120 °C during its industrial production. At the LC deposition onto the grain cut, no domain growth was observed, but the schlieren or thread-like LC texture formed and stayed stable for infinitely long time. The PC film obtained from dissolved grains was dried in a thermobalance. During evaporation, solvent weight *p vs*. time *t* was continuously determined at different stabilized temperatures. After LC deposition onto the surface of the film dried at *T* = 120 °C for the time 10 min < *t* < 15 min, first the thread-like texture occurred and in several hours the textures with entangled thread-likes (Figure 9a) or grain-shaped textures (Figure 9b) formed, depending on the solvent used (dichloromethane or chloroform in the first case and pyridine in the second case). A decrease in the time of the polymer film drying to *t* < 10 min at *T* = 120 °C and the temperature reduction to *T* = 24 °C at all *t* led to the formation of the domain textures shown in Figure 2a–c.

**Figure 10:**
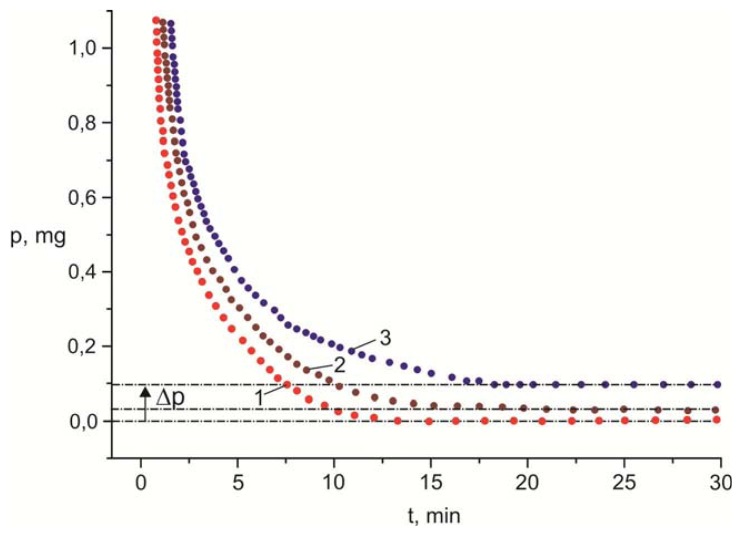
Time dependences of solvent weight *p* in the PC film dried in the thermobalance. Curves 1, 2 and 3 correspond to the temperatures *T* = 120, 50, and 24 °C.

Figure 10 presents the dependences *p* (*t*) for the PC film with the weight *p* = 15 mg deposited by centrifugation onto the glass surface and dried at the temperatures *T* = 120, 50, and 24 °C (curves 1, 2 and 3). For the horizontal portion of curve 1, the value *p* = 0 was taken, since the dependences *p*(*t*) obtained at higher temperatures, up to the glass-transition point *T* = 141 °C, did not differ from this curve within the weight measurement accuracy Δ*p* = ±10 μg. It can be seen that the straight lines *p* = const corresponding to the horizontal portions of the curves *p*(*t*) at *T* = 50 and 24 °C differ from the curve *p*(*t*) = 0 at *T* = 120 °C by the values Δ*p* = 30 and 90 μg. The values of Δ*p* correspond to the amount of the residual solvent in the polymer film after its drying at a chosen temperature.

### 2.5. Memory Effect of the LC Alignment on the PC Surface

**Figure 11:**
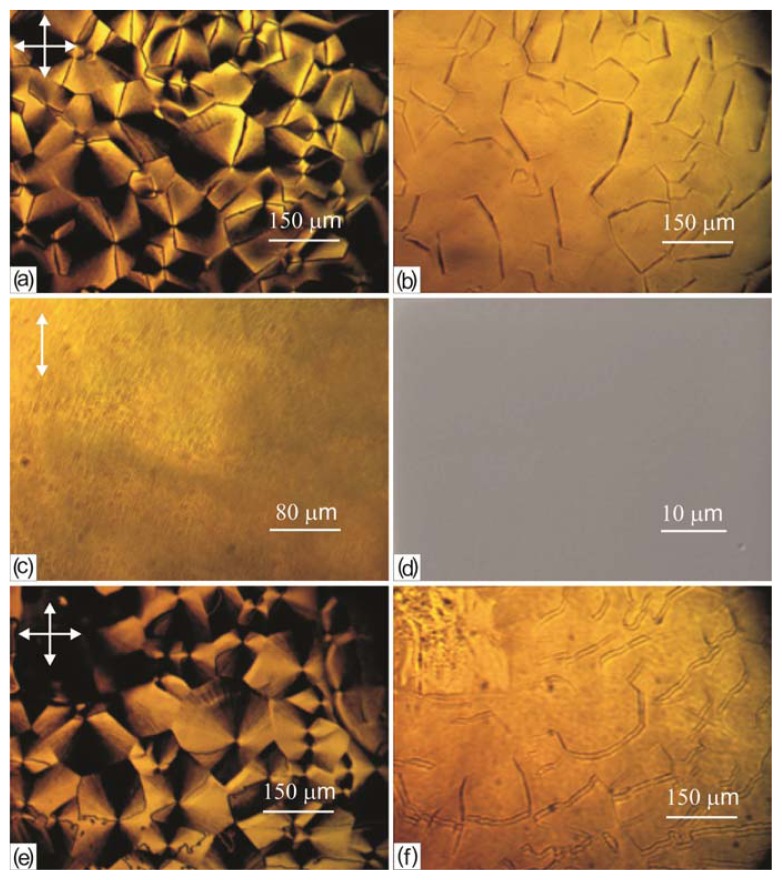
Microphotographs of the 5 CB domain textures on the PC film (**a**,**b**) obtained from the solution in dichloromethane, (**c**,**d**) after LC removal in ethyl alcohol, and (**e**,**f**) after repeated deposition on the PC film. The frames are taken in (**a**–**c**, **e** and **f**) an optical microscope and (**d**) a scanning electron microscope in the topographical mode.

The LC layer with the b-domains was washed in ethyl alcohol, which does not dissolve PC. An optical microscope microphotograph of the layer before washing in crossed polarizers is presented in Figure 11a. Without polarizers, there are pronounced disclination lines on the polymer film surface that have narrow regions near the points corresponding to the domain centers (Figure 11b). After washing, the polymer surface looks as before the LC deposition, without any defects (Figure 11c). Topography of the same surface in a scanning electron microscope is shown in Figure 11d. At the repeated LC deposition onto the washed polymer surface, the domain texture instantly arises again (Figure 11e). However, the textures before and after washing and repeated LC deposition differ from one another. The points on the disclination lines corresponding to the domain centers retain their positions. Meanwhile, the disclination lines change their initial direction and shape: many of them become bent or zigzag. In addition, without polarizers they are observed as double lines (Figure 11f). The difference in the textures in Figure 11a,e can be explained by the fact that the director in the volume LC layer, being perpendicular to the disclination lines, changes its distribution, following them. Thus, the PC surface after the interaction with the LC acquires the memory of the structural ordering modified by the disclination lines.

### 2.6. Structuring the LC Disclination Lines on the PC Surface

**Figure 12:**
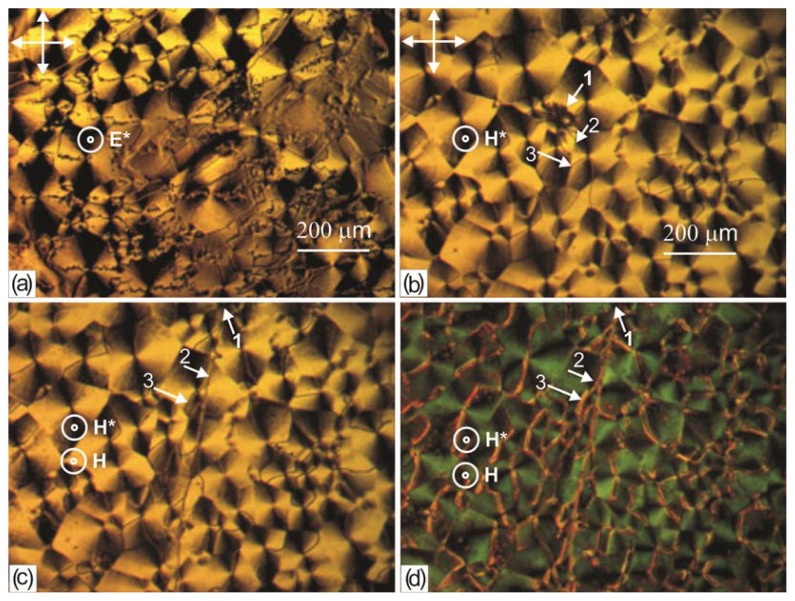
Microphotographs of 5 CB domains formed during the growth on the polymer film obtained from the PC solution in chloroform (**a**) in the electric field E^*^ = 7.5 10^3^ V/cm switched off after the growth finish; (**b**–**d**) in the magnetic field H^*^= 25 kOe switched off after the growth finish; (**b**) without using magnetic field *H*; (**c**) *H* = 1 kOe; (**d**) *H* = 4 kOe.

If during the growth of the nematic domains on the PC surface the LC cell was subjected to mechanical, electrical, or magnetic factors, the disclination lines can appear not localized at the surface. Figure 12 shows a microphotograph of the domain ensemble grown in the electric field (a) or magnetic field (b–d) H* = 25 kOe directed perpendicular to the polymer film. It can be seen that the disclination lines formed a multibranch network, passing through the domain centers. In addition, one can see a ball-shaped defect with the outgoing disclination lines (1) occasionally occurred during the growth. The defect is free, since in the magnetic field *H* = 1 kOe (Figure 12c) it shifted to the image edge. However, the disclination lines appeared in the form of the main line (2) with the outgoing side lines (3) that end at the domain centers. Upon deformation of the volume LC layer by the stronger magnetic field *H* = 4 kOe, the multibranch thread network becomes pronounced (Figure 12d).

## 3. Discussion

Study of the LC domain structures on the PC surface showed that the b-domains are the c-domains with the superimposed planar LC layer. However, from the topological point of view, the c-domains should have a singular point with the force *s* = ± 1 at their centers [2]. Changing the angular position of a domain relative to the crossed polarizers, one can determine the sign of a topological defect. At the rotation of a c-domain in Figure 3, the extinction band positions relative the polarizer directions remain invariable, which corresponds to the sign (+) of the point defect force. This behavior of the domains is characteristic of the schlieren texture; however, in contrast to it, the c-domains with the sign (−) were never observed in our experiments. Thus, we may conclude that the structures under study are not objects of the nematic medium in itself [1,2], but autonomous formations. In this case, the polymer surface should be considered as a source of the domain formation.

One of the possible reasons for the occurrence of domains and disclinations in the LC on the PC surface is the presence of mechanical defects (cracks, protrusions, troughs, *etc*.). The defects can occur under the action of surface tension resulting from diffusion of a free volume during evaporation of the solvent [13], which remains in the film after its drying in air (Figure 10). In addition, one may suggest that the domain ensembles shown in Figure 2b,c are the domain networks with protrusions and troughs, similar to those described in [6–9], in which the LC director configuration is significantly affected by forces of gravity. However, since the domain formation was not affected by the spatial position of the PC substrate and no relief on the polymer surface was found after removal of the LC in optical and scanning electron microscopes (Figure 11c,d), the surface profiling cannot be considered as an origin of the domain formation.

The results of our experiments can neither be treated within the nucleophilic decay of the three-component system LC-polymer-solvent at a constant temperature. First, these components are spatially separated in the sandwich and it is difficult to find the conditions of their intermixing and the phase separation, which were met in the LC structures described in [3–5]. Second, the domain formation in the LC on the PC surface is poorly compatible with the classical concept of nucleation and growth in the LC systems, according to which the time dependence of the diameter of a growing nucleus obeys the universal growth law *d*(*t*) ~ *t*^k^. In this law, growth component *k* in the well-known experiments reported in [11] changes from 0.5 to 1 only near temperature *T*_NI_. At the exponential growth, the value *k* = 1 can be obtained. In our experiment, the time dependence of the diameter of a growing domain (Figure 7) can be described by the universal growth law for the only value *k* = 1. In addition, as can be seen in Figure 5, the domains grow to this size until the neighboring domains limit their propagation.

On the other hand, it can be supposed that the domain formation originates from the occurrence of supramolecular structures on the polymer film surface during evaporation of the solvent, which are visualized by the LC due to the molecular interaction. Since PC is a rigid chain polymer, upon slow evaporation of the solvent from the solution such crystal structures as packs, fibrils, and spherulites can form in it. [14]. Packs and fibrils contain 10–50 polymer chains and are characterized by molecular close packing. Spherulites are tens and hundreds of microns in size and can be seen in a polarizing microscope in crossed polarizers. However, in observation of the PC film (Figure 11c) with the removed structured LC layer in polarized light, the optically dark field is always seen and the crystal polymer structure is not observed. In addition, as follows from Figure 10, evaporation of the solvent from a solution is not slow if the LC film is deposited onto the PC film right after its formation, although in this case no domain formation was observed.

At the same time, the dependence of the domain growth time on temperature and exposure of the polymer film prior to the LC deposition and the different forms of domains depending on the solvent used indicate that the solvent plays an important role in the formation of the LC texture. As is known, in the polymer films there is a surface layer with the chemical structure and density different from those in the volume [13]. This layer forms a potential barrier at the interface preventing solvent output. On the other hand, there exists a well-known phenomenon of extraction of substances in solvents [15]. In particular, in study [16] the authors showed that dichloromethane used by us effectively extracts nematic LC molecules from the droplets emulsified in the polymer matrix. We suppose that extraction of solvent molecules by LC molecules creates the solvent excess in the surface layer and causes the mobility of polymer chains. The amount of the solvent in the thin PC film is not sufficiently large to transfer the polymer surface to the diluted solution state with separate macromolecules. Such a system could be described by methods of statistical physics of volume interactions [17] with the exact analysis of the structural transformations on the polymer surface in the presence of the LC. However, in our case, structuring is the surface of the amorphous PC film with entangled polymer chains. Parts of this surface transfer to the plastic state during the solvent entry. The quantitative analysis of such a system is complicated by the significant mutual effects of statistical factors. Nevertheless, structuring of the PC surface capable of retaining the LC domain configuration can be roughly estimated by energy *E* consisting of characteristic energies *E*_ps_ of the polymer − solvent interaction, energy *E*_pp_ of attraction of polymer links and, additionally, energy *E*_plc_ of the interaction of polymer links with nematic molecules: *E = E*_ps_ + *E*_plc_ − *E*_pp_. To estimate *E*_ps_ of the investigated systems, we calculated the Huggins constants asη_sp_/*c* = [η] + *K*_H_ [η]^2^*c* [14], where *c* is the solution concentration, η_sp_/c = (η − η_0_)/η_0_*c* is the reduced viscosity of the solvent, η and η_0_ are the solvent and solution viscosities, and [η] is the viscosity at the infinite dilution. We measured η_sp_ for different *c* and applied standard calculation technique with extrapolation of the curve η_sp_/*c* to the limit value [18]. Defining [η] from the Staudinger-Kun equation [η] = *KM*^α^ with a value of the molecular *weight M* = 45000 and the coefficients *K*/α = 1.11 × 10^−2^/0.82; 2.77 × 10^−1^/0.5; 2.01 × 10^−2^/0.7 [14], we obtained *K*_H_ = 0.43, *K*_H_ = 0.3, and *K*_H_ = 0.13 for the PC solutions in dichloromethane, chloroform, and pyridine, respectively. Apparently, when dichloromethane or chloroform yielding high values of *K*_H_ and, consequently, high *E*_ps_ are used [14], we have *E*_ps_ + *E*_plc_ >> *E*_pp_ > *kT*. When the amount of the solvent in the surface layer is sufficiently large, then movable parts occur in the polymer chains. On these parts, the *b*- and *c*-domains form. At the same time, when pyridine with low *K*_H_ is used under the same condition, we have *E*_ps_ + *E*_plc_ > *E*_pp_ > *kT*, which is insufficient to strain a part of the polymer chain, which results in the formation of island-shaped domains on the PC surface (Figure 2d). As the amount of the solvent is decreased, e.g., upon drying the polymer film at the temperature *T* = 50 °C (Figure 10), the mobility of the polymer chains drops and the domain formation degrades: randomly formed structural formations with entangled thread visualized by the LC arise on the PC film that are hard to analyze (Figure 9a) or granular textures are observed (Figure 9b). When the solvent is completely removed from the PC film at *T* = 120 °C (Figure 10), the thread-like or schlieren texture forms on the polymer surface.

**Figure 13:**
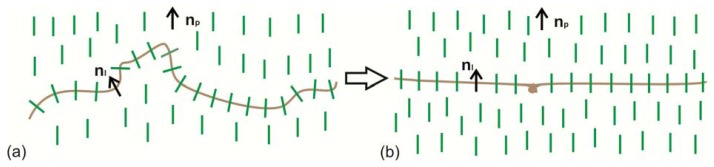
Schematic in plane of the transition of a part of the PC polymer chain with absorbed LC molecules (*L*) from the distorted state (**a**) to the energetically more favorable strengthened state (**b**); n_l_ is the unit vector corresponding to the averaged alignment of absorbed LC molecules along the polymer chain and n_p_ is the director of the planar volume LC layer.

Study of the growth kinetics of the domains located close to one another (Figure 6) or the domain ensembles arising at the repeated deposition of the LC on the structured PC surface (Figure 11e) allows us to make the following assumption. The disclination lines in the domains occur in the presence of structural elements *L* that are the movable parts of the polymer chains with LC molecules absorbed by them perpendicular. The absorbed molecules tend to orient the polymer chains perpendicular to director n_p_ of the planar volume LC layer (Figure 4). The mechanism of absorption of nematic molecules will be investigated in the next work. We denote the averaged alignment of the absorbed molecules along the polymer chain by unit vector n_l_ (Figure 13). Under the action of the local torques from the side of n_p_, element *L* will tend to reduce its free energy. If the chain ends are fixed on the surface, then a more bent element will be found on *L* on which the chain will twist and, owing to this, straighten up. The twisted part will appear an orienting center around which LC molecules will align with the formation of the radial configuration. This process should be slow due to the molecular interactions of the LC and the polymer surface [19,20]. Owing to the absorption of nematic molecules on the PC film, the twist will fix on the surface. At the formation of the structure in an external field, e.g., magnetic, the twist can break away from the surface and pass in the volume (Figure 12). The proposed scenario of the development of the radial configuration (*R*) of the director field n(r) is consistent with the domain growth observed in our experiments.

The occurrence and shape of the disclination lines can be explained by studying the behavior of configurations *L* within *R*. On the texture parts where local director n_r_ and vector n_l_ coincide, lines *L* are indistinguishable. They become visible where n_r_ significantly diverges from n_l_. The divergence should be larger at the periphery of *R* when the consideration is made along *L* and smaller near the *R* center where n_r_ and n_l_ are the closest to one another. Under these conditions, the disclination lines should occur along *L* and they were really observed in the optical microscope. Without polarizers, they looked like wide bands narrowing near their centers (Figure 11b). If under the action of an external factor, a polymer chain part is forced out into the LC layer, the disclination line corresponding to this part looks like a double line (Figure 11f). Thus, the disclination lines arising at the developed configuration *R* and following its radius are the factors visualizing the polymer chain during the domain growth (Figures 5 and 6). Dark defect areas in Figure 6 near the domain centers can be identified as the polymer chain twists visualized by large light scattering due to the sharp change in the refractive index in the strongly strained LC layer. In this figure, one can also see that during the domain growth, molecules of the volume nematic layer do not extend the polymer chains, which could occur due to the anisotropy of the LC surface tension, and only visualize them.

Thus, the domain growth is the gradual radial alignment of LC molecules on the PC surface around the polymer chain twists with the formation of the disclination lines along them. The LC molecules forming the configuration *R* are absorbed on the polymer surface, retaining the surface ordering memory. After removal of the LC layer, the polymer chains are released but the *R* imprint on the PC surface and the twist fixing points remain. At the repeated LC deposition on the PC film, configuration *R* is instantly visualized, but the polymer chains released from the LC form new structural elements *L* aligned in the new direction (Figure 11e). The surface configuration *R* ensures the memory of the planar LC alignment above the domains. The orientation of *L* in the surface polymer layer favors the formation of the stable azimuth direction of nematic director n_p_ in the volume layer.

**Figure 14:**
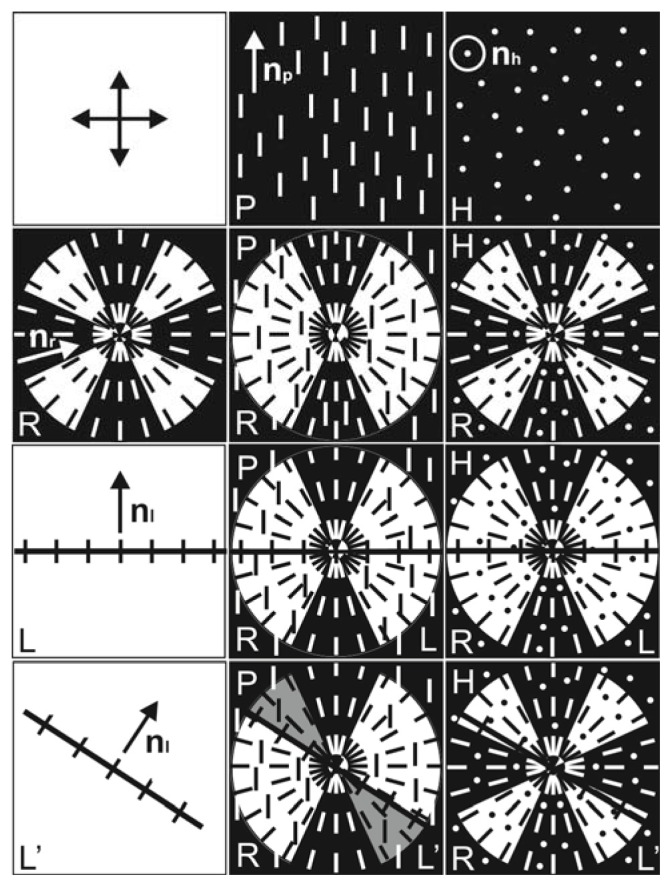
Orientational structures at different configurations of the LC director on the PC surface and in the top volume nematic layer: *P* and *H* are the planar and homeotropic LC orientations in the volume layer, *R* is the radial LC configuration on the PC surface, and *L* and *L′* are the structural elements in the form of polymer chains visualized by LC molecules and located along the direction of one of the polarizers or at an angle of about 45° to it. Combinations of *PR*, *HR*, *PRL*, *HRL*, *PRL′*, *HRL′*, the corresponding domain structures are represented as elements of a matrix with columns containing the orientations *P*, *H* and rows that contain the configurations *R*, *L*, *L′*.

Figure 14 presents the main possible orientational structures at different LC configurations in the volume and on the surface of PC. If the LC alignment in the volume layer is planar (*P*), then at its superposition on the surface configuration *R* with four extinction bands, we will observe the configuration *PR* with two narrow dark bands where the n_p_ and n_r_ orientations are close. In the wide bright regions, these orientations have a large angular divergence. At the superposition of *L* on *PR*, horizontal in the geometry presented in the figure, the configuration *PR* will not change due to the large angular divergence of directors n_p_, n_r_, and n_l_, but *L* will be seen as a dark line against the bright background. If *L* were located vertically, the dark branches in the configuration *PRL* would brighten due to the large difference between the n_r_ and n_l_ orientations and the entire configuration disk would become bright. However, since n_p_ and n_l_ tend to orient perpendicular to one another, this configuration is not shown in the figure and the bright domains were not observed in our experiments. The presented configuration *PRL* corresponds to the *b*-domain shown in Figure 3a. If *L* makes a certain angle with the light polarization directions (*L′*), then the orientational divergence between n_p_ and n_l_ decreases and brightened sectors will appear in the configuration *PRL′*. The configuration *PRL′* corresponds to the b-domain shown in Figure 3c. If in the volume layer the LC has the homeotropic alignment (*H*), then at the superposition of the latter on the surface configuration *R* with four extinction bands, the configuration *HR* will also be seen with narrow dark bands. At the superposition of *L* on *HR*, vertical or horizontal in the geometry presented in the figure, the dark bands through which *L* passes will brighten due to the large angular divergence of directors n_r_ and n_l_, and *L* will be visualized as a dark line against the bright background. The configuration *HRL* corresponds to the c-domain shown in Figure 3f. In the configuration *HRL′*, the bright regions will remain due to the large angular divergence between n_r_ and n_l_, but *L* will be seen as a dark line against the bright background. The configuration *HRL′* corresponds to the *c*-domain shown in Figure 3d. The orientation change *P*→*H* should lead to the configuration transformation *PRL*→*HRL*. This transformation can occur at the growing effect of the oblique alignment of the nematic on the free droplet surface on the planar LC alignment on the PC surface because of a decrease in the thickness of the volume nematic LC layer at approaching the droplet periphery, similar to the LC cell with the homeoplanar orientation [21]. The domain ensembles in Figure 2b,c correspond to the considered transformation. The transformation *PRL*→*HRL* can occur also at the temperature variation (Figure 8). The orientational transition can be caused by different temperature dependences of the polar energies of the nematic anchoring to movable polymer chains *W*_L_ and radially absorbed LC molecules with polymer surface *W*_R_.

## 4. Experimental Procedure

We investigated the well-known nematic LCs 4-metoxybenzylidene-4′-butylaniline (MBBA) [22] and 4-n-pentyl-4′-cyanobiphenyl (5CB) [23] (both from Merck, Darmstadt, Germany) with the phase transition sequences *Cr*-22°C-*N*-47°C-*I* and *Cr*-21.5°C-*N*-35°C-*I*, respectively. Rigid chain polymer polycarbonate (PC) was used as an orienting surface. The polymer PC is a granular material synthesized from bisphenol A [14].

whose groups are coupled with carbonate groups in a polymer chain. We applied Poly (Bisphenol A carbonate) with average molecular weight M ~45000 from Sigma-Aldrich Co. LLC (St. Louis, MO, USA). Polycarbonate grains were dissolved in the solvents with different solvations in PC: dichloromethane (CH_2_Cl_2_), chloroform (CHCl_3_), or pyridine (C_5_H_5_N) in concentration of 2%. We applied the dichloromethane stabilized with ~20 ppm of amylene, chloroform stabilized with ~150 ppm of amylene and pyridine, 99% PS from Panreac Quimica S.L.U (Barcelona, Spain). The polymer solutions were deposited onto a smooth clean glass surface by centrifugation or flowing. At the solvent evaporation for several seconds with average evaporating rate 40%/min, a PC film formed on the glass surface. The film thickness was from 100 nm using centrifugation to few microns when applying the method of flowing. The thickness of film was measured using a device Filmetric F50-UVX (San Diego, CA, USA) up 0.4%. The PC film IR spectrum was measured by a Vertex-80v Fourier-transform spectrometer (Bruker Optik GmbH, Ettlingen, Germany). The nematic LC was deposited onto the surface of the dried polymer film in the form of a droplet or a thin layer in the cell with a cover glass. The obtained sandwiches were studied using a Carl Zeiss LMA-10 polarizing microscope (Oberkochen, Germany). The polymer film surface was tested in Hitachi S-5500 (Hitachinaka, Japan) scanning electron microscope in topographical mode. The temperature of the samples preparation and investigations was *T* = 24 °C, except for the experiments with the orientational transformations in the nematics. A dc magnetic field up to *H* = 25 kOe was generated by an electromagnet.

## 5. Conclusions

We investigated the domain structures occurring in the nematic liquid crystals deposited onto the surface of a polycarbonate film obtained with the use of solvents with different solvations. During the growth, the domain formation against the background of the initial tread or schlieren texture was established; radially symmetrical structures with two and four extinction bands were observed, depending on thickness of the volume nematic layer in a liquid crystal droplet. We found the temperature-induced transition between the domain structures caused by the director reorientation from the planar to homeotropic state in the liquid crystal volume. We demonstrated that the solvent plays an important role in the domain formation: extraction of solvent molecules by liquid crystal molecules leads to a solvent excess in the polymer surface layer and causes the mobility of the polymer chains. We demonstrated the LC structural ordering memory effect caused by absorption of nematic molecules forming the radial configuration of the director field on the PC surface and the disclination lines passing through the domains along their radii. We established that the disclination lines orient perpendicular to the director of the planar volume LC layer in the substrate plane. We showed that the disclination lines result from the interaction of LC molecules with movable parts of the polymer chains. The observed textures were schematically compared with different combinations of the LC volume orientations and the radial distribution of the director field and the disclination lines at the PC surface.
